# TNF-Like Weak Inducer of Apoptosis Aggravates Left Ventricular Dysfunction after Myocardial Infarction in Mice

**DOI:** 10.1155/2014/131950

**Published:** 2014-02-20

**Authors:** Kai-Uwe Jarr, Sabine Eschricht, Linda C. Burkly, Michael Preusch, Hugo A. Katus, Norbert Frey, Emmanuel Chorianopoulos

**Affiliations:** ^1^Department of Cardiology, Angiology and Pulmonology, Heidelberg University Hospital, Im Neuenheimer Feld 410, 69120 Heidelberg, Germany; ^2^Department of Immunology, Biogen Idec, Cambridge, MA 02142, USA; ^3^Department of Cardiology and Angiology, University of Kiel, Schittenhelmstraße 12, 24105 Kiel, Germany

## Abstract

*Background*. TNF-like weak inducer of apoptosis (TWEAK) has recently been shown to be potentially involved in adverse cardiac remodeling. However, neither the exact role of TWEAK itself nor of its receptor Fn14 in this setting is known. *Aim of the Study*. To analyze the effects of sTWEAK on myocardial function and gene expression in response to experimental myocardial infarction in mice. *Results*. TWEAK directly suppressed the expression of PGC-1**α** and genes of oxidative phosphorylation (OXPHOS) in cardiomyocytes. Systemic sTWEAK application after MI resulted in reduced left ventricular function and increased mortality without changes in interstitial fibrosis or infarct size. Molecular analysis revealed decreased phosphorylation of PI3K/Akt and ERK1/2 pathways associated with reduced expression of PGC-1**α** and PPAR**α**. Likewise, expression of OXPHOS genes such as atp5O, cycs, cox5b, and ndufb5 was also reduced. Fn14 −/− mice showed significantly improved left ventricular function and PGC-1**α** levels after MI compared to their respective WT littermates (Fn14 +/+). Finally, inhibition of intrinsic TWEAK with anti-TWEAK antibodies resulted in improved left ventricular function and survival. *Conclusions*. TWEAK exerted maladaptive effects in mice after myocardial infarction most likely via direct effects on cardiomyocytes. Analysis of the potential mechanisms revealed that TWEAK reduced metabolic adaptations to increased cardiac workload by inhibition of PGC-1**α**.

## 1. Introduction

Tumor necrosis factor- (TNF-) like weak inducer of apoptosis (TWEAK, also TNFsf 12, Apo3L) is a member of the TNF family of cytokines with multifunctional properties [1, 2]. Although inflammatory cytokines and their receptors are considered to play a crucial role in cardiac remodeling and may promote both acute heart failure and progression to chronic myocardial dysfunction, attempts to inhibit the action of inflammatory cytokines have shown conflicting results in patients with heart failure [3–10].

TWEAK has been described as an angiogenic factor and as a regulator of myoblast differentiation as well as atrophy and tumor apoptosis [11–15]. FGF-inducible-14kD-protein (Fn14, also known as TNFrsf12a or TWEAKR) is currently the only known signaling receptor for TWEAK, and TWEAK is the only known ligand for Fn14. However, Fn14 may be activated independently of TWEAK binding by homopolymerization, indicating a potential ligand-independent function of Fn14 [13]. Fn14 is the smallest member of the TNF-receptor family, a type 1 transmembrane protein of 102 amino acid length, and is expressed at relatively low levels in normal tissue but highly inducible in response to tissue injury [16].

Recently, TWEAK and its receptor Fn14 have been described to be potentially involved in cardiac pathology [17, 18]. TWEAK transgenic animals develop dilated cardiomyopathy and heart failure [18]. We and others have recently reported upregulation of the TWEAK-Fn14 axis in the remodeling heart after myocardial infarction in mice and rats [17, 19]. In addition, we have recently shown that elevated sTWEAK levels predicted short-term prognosis in patients with acute ST-elevation myocardial infarction (STEMI) [20]. The exact role of TWEAK in this setting is still unknown; however, inhibition of the metabolic master regulator PGC-1*α* has been proposed as potential mechanism [21, 22].

We therefore aimed to further investigate whether activation of the TWEAK-Fn14 axis exerted any effect on myocardial function and remodeling in mice subjected to experimental myocardial infarction. Finally, we explored the possibility that inhibition of TWEAK-Fn14 may protect mice against adverse remodeling after myocardial infarction.

## 2. Materials and Methods

### 2.1. Animals and Myocardial Infarction Model

Male CD-1/Swiss mice of 2-3 months of age underwent induction of myocardial infarction. Myocardial ischemia was induced by ligation of the left anterior descending (LAD) coronary artery. Briefly, mice were intubated and ventilated with 2% isoflurane (Minivent, Hugo-Sachs, March-Hugstetten, Germany). After a left-sided thoracotomy, the left anterior descending coronary artery was occluded by a permanent ligation (8–0 suture, Ethicon, Norderstedt, Germany). Myocardial ischemia was affirmed by pale decolorisation of the depending myocardium. Sham-operations included all procedures except ligation of the LAD. The experiment conforms with the Guide for the Care and Use of Laboratory Animals published by the U.S. National Institutes of Health under Institutional Protocol numbers G170/08;G119/12;G121/12;G174/08. Recombinant human TWEAK (PeproTech, Hamburg, Germany) was injected every 3 days i.p. over 4 weeks after MI starting on day 1 after LAD ligation at a dose of 200 *μ*g/kg body weight. Fn14 −/− mice, neutralizing monoclonal antibodies against TWEAK, and control IgG (Biogen Idec) were generated as described [23, 24]. Injection of antibodies was performed every 3 days i.p. over 4 weeks, with the first dose injected prior to LAD ligation at a dose of 10 mg/kg body weight. For mRNA and protein analysis the nonischemic myocardium, remote from the infarcted area, was separated and analyzed. For histological analyses hearts were snap frozen in liquid nitrogen, cut, and then fixed in 4% paraformaldehyde. Masson's trichrome staining for fibrosis and infarct size was performed with the trichrome staining kit (Sigma, München, Germany). Morphometric analyses were performed using ImageJ analysis software.

### 2.2. Functional Analysis

Echocardiography of mice was carried out using a Hewlett Packard Sonos 5500 Ultrasound system with a 12 MHz transducer. Three independent M-mode measurements per animal were obtained by 2 independent blinded examiners. Echocardiographic data were achieved at heart rates between 450 and 550 beats/min, respectively. The following parameters were obtained in a short axis at the level of the papillary muscles: end systolic and end diastolic chamber diameter and left ventricular fractional shortening {FS% = (LVEDD − LVESD)/LVEDD × 100}. Invasive assessment of cardiac hemodynamics: briefly, we anesthetized mice with using 4% isoflurane (v/v) supplemented with oxygen, intubated, and artificially ventilated them by a custom-designed mouse ventilator (Minivent, Hugo Sachs, March-Hugstetten, Germany). The mechanical ventilation was maintained using 2% isoflurane (v/v) supplemented with oxygen. After insertion into the left main carotid artery the hemodynamic catheter was advanced retrogradely in the left ventricle using a 1 F pressure-loop catheter (Millar Instruments, Houston, USA). In vivo hemodynamic parameters were obtained with LabChart analysis software (ADInstruments, Spechbach, Germany).

### 2.3. Isolation and Culture of Cardiomyocytes

1-2-day old Wistar rats (Charles River) were decapitated and hearts were harvested and minced in ADS. Subsequently, up to six digestion steps were carried out with pancreatin (Sigma, 0.6 mg/mL) and collagenase type II (Worthington, Lakewood, USA, 0.5 mg/mL) in sterile ADS buffer containing 120 mmol/l NaCl, 20 mmol/l HEPES, 8 mmol/l NaH_2_PO_4_, 6 mmol/l glucose, 5 mmol/l KCl, and 0.8 mmol/l MgSO_4_, pH 7.4. Neonatal rat ventricular cardiomyocytes (NRVCMs) were purified from contaminating fibroblasts using a Percoll (Amersham Biosciences) gradient centrifugation step. Finally, NRVCMs were resuspended and cultured in Dulbecco's modified Eagle's medium (DMEM) containing 10% FCS, penicillin/streptomycin, and L-glutamine (all from PAA). Adult rat ventricular cardiomyocytes (ARVCMs) were isolated from Sprague-Dawley rats (~300 g) using the collagenase digestion method. ARVCMs were plated on laminin-coated dishes. ARVCMs were cultured in a HEPES-modified medium 199 (M199, Sigma S7528, supplemented with 5 mM  taurine, 5 mM  carnitine, 5 mM  creatine, 5 mM  N-mercaptopropionyl glycine, 0.1 *μ*M insulin, 10,000 U/mL penicillin, and 10 mg/mL streptomycin, pH 7.25).

### 2.4. Cell Culture Experiments

Human recombinant TWEAK was from PeproTech (Hamburg, Germany). All experiments were performed under serum-free conditions. Cells were then either left untreated or treated with 10–100 ng/mL TWEAK alone for various time periods. Cell culture experiments for RNA isolation were performed for 6 hours and those for protein isolation after 24 hours, if not indicated otherwise. Cardiomyocytes were transfected with replication deficient adenoviral vectors carrying TNFSF12 (Vector BioLabs, Philadelphia, USA) or LacZ at a multiplicity of infection (MOI) of 10 and 50. Analyses were performed after 48 hours of adenoviral infection. The ADP/ATP ratio was measured after 6 hours according to the manufacturer's instructions (EnzyLight ADP/ATP Ratio Assay Kit, ELDT-100, Bioassay Systems, Hayward, USA). Metabolic experiments and ADP/ATP assay kit as functional readout were performed in both NRVCMs and ARVCMs since immature and mature cardiomyocytes differ in terms of metabolism, gene expression, and receptor composition. Total RNA was isolated using the TRIzol method (Invitrogen, Karlsruhe, Germany) according to the manufacturer's instructions and resuspended in DEPC-treated water (Sigma, München, Germany). RNA integrity and purity were verified by measurement of OD260/OD280 absorption ratios. TUNEL assay (Roche Cell death detection kit) was performed after 24 hours.

### 2.5. Quantitative Real-Time PCR

DNase I-digested total RNA of each condition was transcribed into cDNA using the Superscript III first strand kit (Invitrogen, Karlsruhe, Germany). 18S rRNA served as an internal standard. For quantitative real-time PCR, the Platinum SYBR Green qPCR SuperMix-UDG system (Invitrogen, Karlsruhe, Germany) was used in the ABI Prism 7000 Sequence DetectionSystem (Applied Biosystems, Foster City, USA). Each PCR amplification step was carried out using the following 5 conditions: 2 minutes at 95°C, followed by a total of 40 temperature cycles (15 seconds at 95°C, 15 seconds at 57°C, and 1 minute at 72°C). Specificity of the reactions was confirmed by performing a dissociation protocol after each cycle and comparison of the results with the expected melting-point temperature of the amplicon as well as by verification of the expected size of the product on a 2% agarose gel. The relative expression levels of these genes were calculated by the ddCT method with normalization to 18S expression. Primers used for quantitative RT-PCR are listed in Supplementary Table 1 (available online at http://dx.doi.org/10.1155/2014/131950).

### 2.6. Immunoblotting

Cardiomyocytes were harvested and lysed in completed RIPA buffer containing 10 mmol/l Tris, 15 mmol/l EDTA, pH 7.5, 1% NP 40 (v/v), 0.5% sodium deoxycholate (w/v), 0.1% SDS (w/v) (Sigma, München, Germany), protease inhibitor cocktail tablets (Roche, Rotkreuz, Switzerland), and phosphatase inhibitor cocktails (Sigma, München, Germany). After up to three brief freeze-and-thaw cycles and a centrifugation step whole cell lysate was obtained. Heart samples of animals were harvested, immediately transferred into completed RIPA buffer containing protease inhibitor cocktail tablets (Roche, Rotkreuz, Switzerland), as well as phosphatase inhibitor cocktails (Sigma, München, Germany), and homogenized using an Ultra-turraxTM tissue separator (Janke & Kunkel, Staufen, Germany). Equivalent amounts of protein were subjected to sodium dodecyl sulfate- (SDS-) polyacrylamide gel electrophoresis (PAGE) and transferred to PVDF-membrane by electroblotting. The membrane was probed with respective primary antibodies at a dilution according to the manufacturer's protocol. Application of the primary antibody was followed by incubation with a horseradish peroxidase-coupled secondary antibody (1 : 10.000, Santa Cruz Biotechnology, Heidelberg, Germany). Visualization was achieved using a chemiluminescence kit (ECL detection, Amersham Biosciences, Freiburg, Germany). Antibodies used are listed in Supplementary Table 2.

### 2.7. Statistical Analysis

All results are shown as the mean ± standard error of the mean (SEM) unless stated otherwise. Testing was performed by the Student *t*-test or one-way ANOVA. Nonparametric tests like Mann-Whitney *U* test or Kruskal-Wallis test were used when variables were not tested or normally distributed. Chi-square test was used to test categorical variables. Values of *P* < 0.05 were considered significant.

## 3. Results

### 3.1. TWEAK Inhibits PGC-1*α* in Cardiomyocytes and Reduces OXPHOS Gene Expression

**Figure 1:**
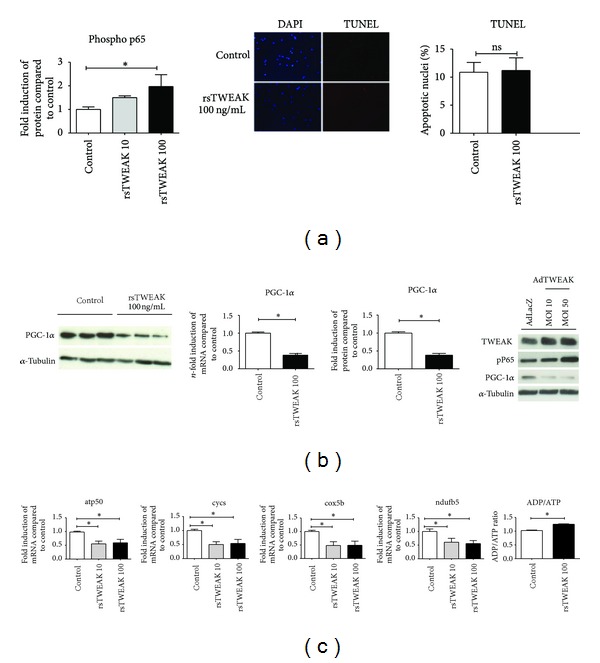
TWEAK directly promotes metabolic maladaptation by PGC-1*α* and OXPHOS gene inhibition in cardiomyocytes. (a) TWEAK dose dependently phosphorylated p65 without affecting cardiomyocyte apoptosis. TUNEL assay (b). Recombinant sTWEAK directly inhibited PGC-1*α* on mRNA and protein level in cardiomyocytes. Similar results could be observed by using an adenoviral vector containing TWEAK as an insert. (c) Likewise, OXPHOS genes like atp5O, ndufb5, cycs, and cox5b were also dose dependently inhibited by recombinant sTWEAK, which resulted in an overall increased ADP/ATP ratio (*n* = 4 for each experimental group).

TWEAK is a known activator of NF-kB in cardiomyocytes [17]. NF-kB activation in turn is known to affect expression of PGC-1*α*, a master regulator of myocardial metabolism [25]. Increased dose-dependent phosphorylation of p65 indicated activation of NF-kB in the absence of increased apoptosis as assessed by TUNEL staining (Figure 1(a)). Recombinant soluble TWEAK (rsTWEAK) directly reduced PGC-1*α* expression on mRNA and protein level. Similar results could be observed, when cardiomyocytes were treated with an adenoviral vector expressing TWEAK (Figure 1(b)). In addition, reduced expression of genes involved in oxidative metabolism (OXPHOS) was observed, when cardiomyocytes were treated with rsTWEAK (Figures 1(b) and 1(c)). As a consequence, ADP/ATP ratio was elevated in cardiomyocytes treated with rsTWEAK (Figure 1(c)).

### 3.2. Expression of TWEAK and Fn14 in the Remote Myocardium after Experimental Myocardial Infarction

**Figure 2:**
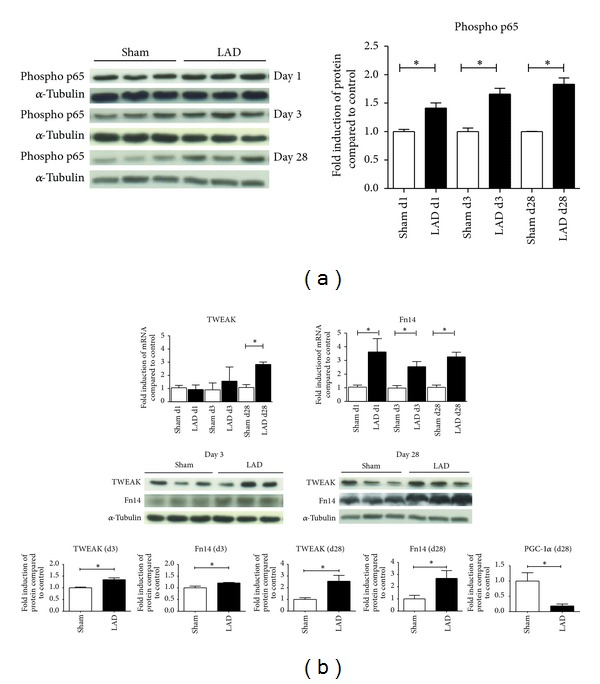
Activation of NF-kB pathway and expression of TWEAK and Fn14 in the remote myocardium after MI. (a) Progressive activation of NF-kB signaling demonstrated by increased phosphorylation of p65 could be observed in the remote myocardium of mice after LAD ligation. Representative western blots (*n* = 5 for sham; *n* = 6 for LAD at each time point). (b) RT-PCR analysis of TWEAK and its receptor Fn14 revealed that both were upregulated in the remote myocardium. However, whereas Fn14 expression occurred rapidly after MI in the remote zone and remained consistently elevated, TWEAK expression increased later. Western blot analyses for TWEAK and Fn14 in the remote myocardium confirmed these findings (*n* = 5 for sham; *n* = 6 for LAD at each time point). Likewise we observed reduced expression of PGC-1*α* in the failing myocardium after MI.

Mice subjected to experimental infarction due to LAD ligation developed progressive left ventricular dysfunction with all functional and neurohumoral signs of heart failure during 4-week follow-up (Supplementary Figure 1). Within days, activation of NF-kB indicated by phosphorylation of p65 was evident in the nonischemic remote myocardium (Figure 2(a)). RT-PCR and protein analysis of TWEAK and Fn14 expression revealed early and persistent temporal expression of TWEAK and Fn14 up to 28 days after induction of myocardial infarction (MI) (Figure 2(b)). Both Fn14 and TWEAK protein were subsequently increased in the remote remodeling myocardium during the first 28 days after MI (Figure 2(b)), indicating a prolonged activation of the TWEAK-Fn14 axis in the remote nonischemic myocardium. In addition, PGC-1*α* mRNA levels 28 days after MI were significantly reduced in the remote myocardium (Figure 2(b)).

### 3.3. Soluble TWEAK Promotes Left Ventricular Dysfunction and Mortality after Experimental Myocardial Infarction

**Figure 3:**
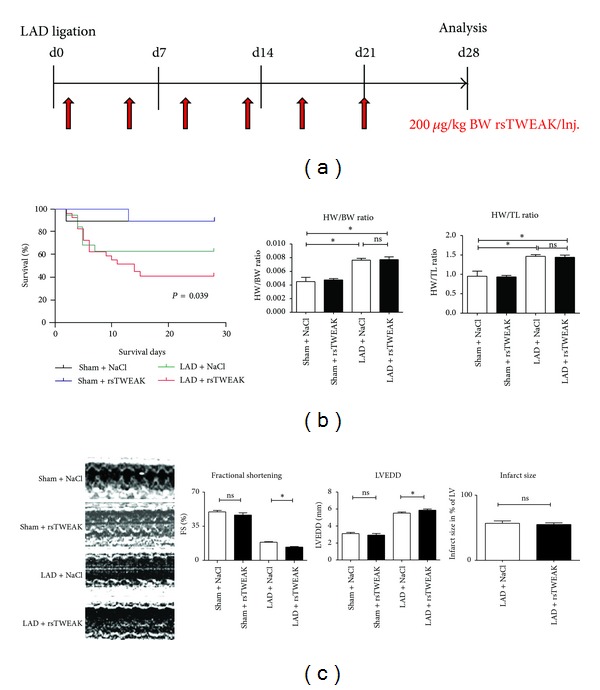
Deleterious effects of recombinant sTWEAK on myocardial function and survival after MI. (a) Recombinant sTWEAK (or vehicle (NaCL)) was injected every 3 days i.p. at a dose of 200 *μ*g/kg body weight for each injection starting 1 day after induction of myocardial infarction in mice. (b) Treatment of mice with recombinant sTWEAK for 28 days resulted in significantly increased mortality after myocardial infarction compared to control animals (*P* = 0.039 sTWEAK versus NaCL). No changes in the degree of myocardial hypertrophy could be observed. (c) Echocardiographic analysis revealed reduced left ventricular function and increased left ventricular end-diastolic diameters (LVEDD) (both *P* < 0.05; sham + NaCL *n* = 4; sham + rsTWEAK *n* = 6; LAD + NaCL *n* = 12; LAD + rsTWEAK *n* = 20).

We further analyzed which influence sTWEAK had on left ventricular function after MI. Animals subjected to LAD ligation were repetitively injected with rsTWEAK at a dose of 200 *μ*g/kg body weight per injection for up to 28 days (Figure 3(a)). No differences in early perioperative (<7 days after ligation of the LAD) mortality after LAD ligation were found. However, there was a significantly increased late (>7 days after ligation) mortality in mice treated with rsTWEAK (*P* = 0.039) (Figure 3(b)). Necropsy studies did not reveal any specific reason for the late mortality in the rsTWEAK group, especially no signs of late ventricular rupture in TWEAK-treated animals with the doses used.

Structural analysis of the remote myocardium also revealed no differences in heart weight/body weight ratios (HW/BW) or in heart weight/tibia length ratios (HW/TL) between rsTWEAK- and NaCL-treated animals (Figure 3(b)). Compared to control animals, rsTWEAK-treated mice showed no significant changes in infarct size (Figure 3(c)). Left ventricular function, however, was further reduced in rsTWEAK-treated mice (*P* < 0.001; Figure 3(c)). Increased end-diastolic diameters revealed incremental cardiac chamber dilation (*P* < 0.05; Figure 3(c)). At the dose used for injection of rsTWEAK, no impairment of left ventricular performance or increased mortality was observed in sham operated mice (see also Figures 3(b) and 3(c)).

### 3.4. TWEAK-Induced Ventricular Maladaptation without Affecting Interstitial Fibrosis

**Figure 4:**
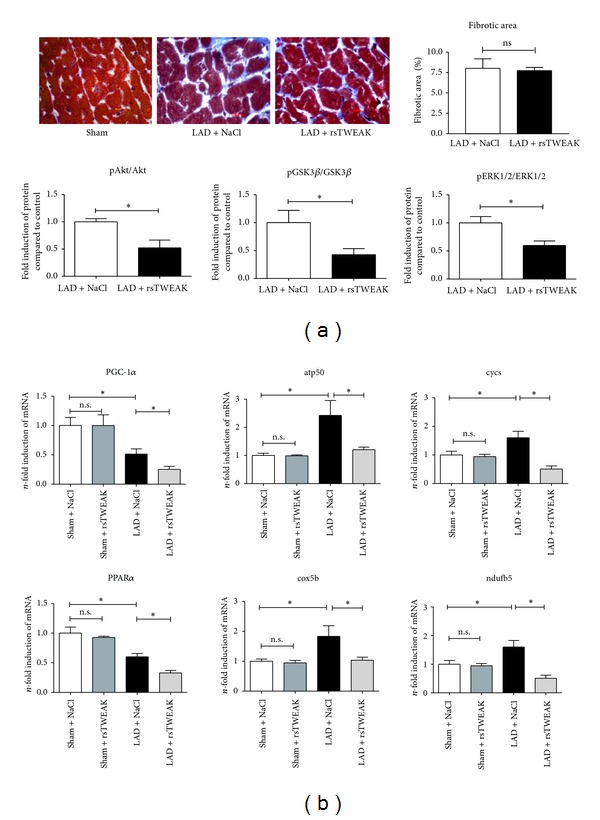
sTWEAK promotes metabolic maladaptation in the remote myocardium after MI. (a) No significant changes in infarct size or interstitial fibrosis were noted in rsTWEAK-treated mice. Phosphorylation of Akt, GSK3*β*, and ERK1/2 was significantly reduced in the myocardium of TWEAK-treated animals compared to vehicle-treated mice (*n* = 5 for NaCL and *n* = 4 for rsTWEAK). (b) PGC-1*α* and PPAR*α* expression in the remote myocardium after MI exhibited greater reductions when mice were treated with rsTWEAK. Likewise, expression of genes involved in oxidative metabolism (OXPHOS) like atp50, cycs, ndufb5, or cox5b was inhibited by treatment of animals with rsTWEAK (*n* = 5 for sham + NaCL, sham + TWEAK and LAD + NaCL, and LAD + rsTWEAK).

The degree of interstitial fibrosis at 28 days after MI was similar in rsTWEAK-treated mice and control (NaCL) mice (Figure 4(a)). No evidence for increased apoptosis was found in the hearts of mice with rsTWEAK-treatment (less than 1 apoptotic nucleus in 1000 nuclei counted). Compared to control mice, animals treated with rsTWEAK did show not only reduced myocardial activation of PI3K /Akt, but also reduced levels of ERK1/2 phosphorylation in the remote myocardium (Figure 4(a)).

Based on our findings in cardiomyocytes, we examined PGC-1*α* expression during treatment with rsTWEAK at day 7 after LAD ligation and found that rsTWEAK-treated mice showed a greater reduction in expression of metabolic master regulators PGC-1*α* and PPAR*α* (Figure 4(b)). Likewise, expression of genes involved in oxidative phosphorylation (OXPHOS), like atp5O, cycs, ndufb5, or cox5b, was reduced in ventricles of rsTWEAK-treated mice consistent with aggravation of maladaptive left ventricular remodeling after MI.

### 3.5. Inhibition of the TWEAK/Fn14 Improves Left Ventricular Function after Myocardial Infarction

**Figure 5:**
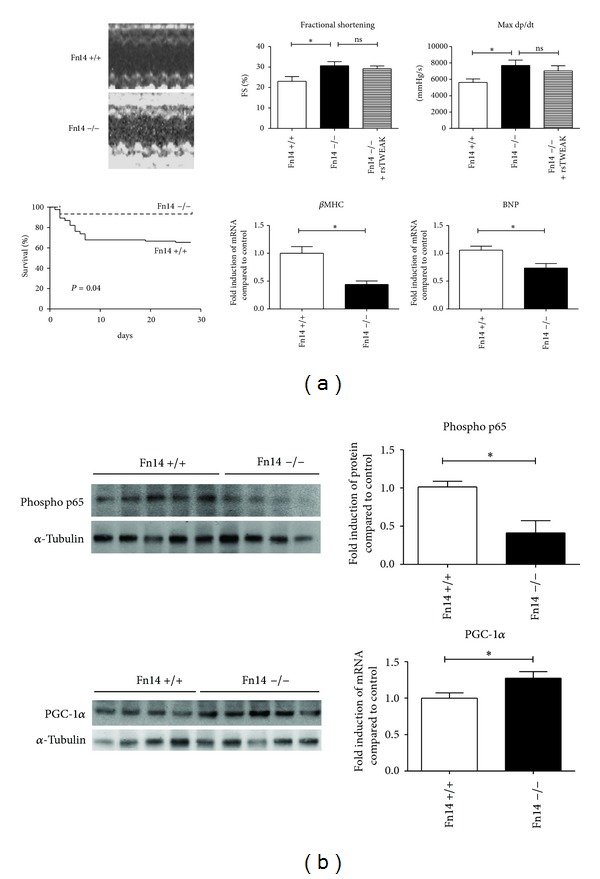
Genetic ablation of the Fn14 improves ventricular dysfunction and survival. (a) Genetic ablation of Fn14 in Fn14 −/− mice protected from progressive deterioration of left ventricular function after MI. Overall this resulted in improved survival after MI (*P* = 0.04; *n* = 15 for each group for survival analysis). (b) Fn14 −/− mice also demonstrated reduced phosphorylation of p65. In addition upregulation of PGC-1*α* compared to wildtype littermates (Fn14 +/+) was also observed (*n* = 5 for Fn14 +/+ and *n* = 6 for Fn14 −/− for molecular analysis).

We next tested whether genetic ablation of the TWEAK-Fn14 axis had beneficial effects on myocardial function after MI. At baseline, fractional shortening in Fn14 −/− mice was not different compared to their respective wildtype littermates (Fn14 +/+) (*P* = 0.9578). Myocardial infarction was induced in Fn14 −/− mice and functional, histological, and molecular parameters were compared to their wildtype littermates. Fn14 −/− mice showed improved left ventricular function on hemodynamic and echocardiographic evaluation and were resistant to the harmful effects of rsTWEAK on left ventricular function (Figure 5(a)). Fn14 −/− mice further displayed reduced phosphorylation of p65 and preserved PGC-1*α* levels compared to their wildtype littermates (Figure 5(b)). Of note, Fn14 −/− mice also revealed improved survival (*P* = 0.04, Figure 5(a)).

**Figure 6:**
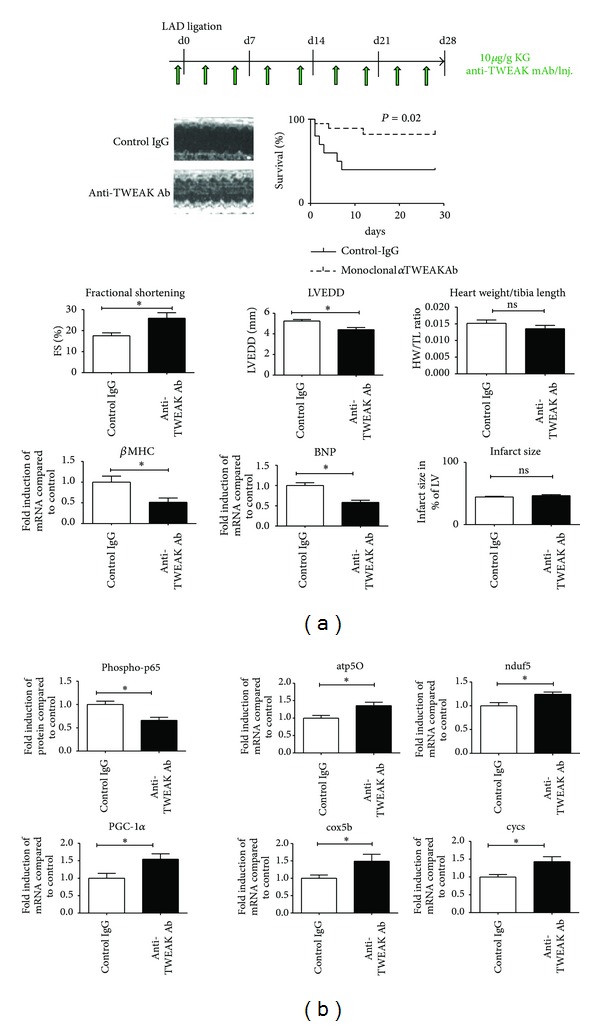
Inhibition of intrinsic TWEAK activity improves survival after myocardial infarction in mice. (a) CD-1 mice were treated with neutralizing monoclonal antibodies against TWEAK or control IgG for 4 weeks after myocardial infarction. Anti-TWEAK treatment resulted in improved survival, preserved left ventricular function, and reduced expression of *β*-MHC and BNP. These effects were independent of infarct size and the degree of left ventricular hypertrophy. (b) Anti-TWEAK treatment reduced the phosphorylation of p65 in the remote myocardium and preserved the expression of PGC-1*α* and OXPHOS genes like atp5O, ndufb5, cycs, and cox5b (molecular analysis *n* = 6 for each group; functional analysis *n* = 12 for each group).

Finally, we tested whether inhibition of intrinsic TWEAK-Fn14 signaling after myocardial infarction may be cardioprotective. Therefore infarcted animals were either treated with neutralizing monoclonal antibodies against TWEAK (anti-TWEAK mAb) or with control IgG, both started one day prior to LAD ligation. Treatment of mice with anti-TWEAK mAb resulted in significantly improved survival after myocardial infarction (Figure 6(a)). Echocardiography and invasive hemodynamics revealed significantly improved left ventricular function and decreased end-diastolic diameters in anti-TWEAK treated mice compared to control animals despite similar infarct sizes (Figure 6(a)). As expected, no changes in heart weight/body weight ratio (HW/BW) or heart weight/tibia length (HW/TL) ratio were found (Figure 6(a)). In line with our findings in knockout mice, anti-TWEAK-treated mice showed reduced activation of the NF-kB pathway and increased levels of PGC-1*α* and genes of oxidative metabolism (OXPHOS) (Figure 6(b)).

## 4. Discussion

Here we report for the first time the effects of sTWEAK and anti-TWEAK strategies after experimental myocardial infarction in mice. Animals treated with sTWEAK after induction of myocardial infarction developed worsened left ventricular function and showed enhanced late mortality compared to control mice. These TWEAK-induced effects were independent of changes in the extent of fibrosis and interstitial remodeling but most likely mediated through direct metabolic effects via inhibition of PGC-1*α* in cardiomyocytes. In turn, inhibition of the TWEAK-Fn14 axis by either genetic ablation of its receptor Fn14 or treatment of mice with anti-TWEAK antibodies improved left ventricular dysfunction after MI.

We have recently reported that levels of sTWEAK were increased in patients with ST-elevation myocardial infarction (STEMI) and independent predictors of an adverse short-term prognosis [20]. Although transgenic overexpression of TWEAK in mice has been reported to induce dilated cardiomyopathy with increased interstitial fibrosis, it elongated cardiomyocytes and ventricular dilation after a few months [18]. However, it remains unclear whether the TWEAK-Fn14 axis plays a role in the setting of myocardial infarction.

Recent evidence suggested direct effects of sTWEAK on cardiomyocytes [17, 21]. We and others have reported rapid stress-induced upregulation and functional relevance of Fn14 in cardiomyocytes [17, 26]. Shi et al. further reported metabolic effects of transgenic overexpression of TWEAK through inhibition of PGC-1 alpha [21]. Activation of NF-kB is known to cross talk with many different pathways including the Akt and PGC-1*α* pathways, which are central for compensating myocardial stress [25, 27]. In this context we found that TWEAK treatment after MI resulted in an overall reduced activation of different protective mediators of cardiac hypertrophy like Akt, GSK3*β*, or ERK1/2.

Recombinant sTWEAK further reduced the levels of PGC-1*α* in failing hearts after MI. Conversely, either genetic ablation of the TWEAK-receptor Fn14 in Fn14 −/− mice or inhibition of TWEAK via neutralizing antibodies resulted in improved left ventricular function compared to control animals and preserved PGC-1*α* levels in failing hearts. The findings suggest a role for TWEAK/Fn14 in the crosstalk between the cytokine system and metabolism at least in mice. PGC-1*α* is dynamically regulated and key in the regulation of metabolic adaptations to heart failure [28, 29].

It is possible that longer treatments with rsTWEAK would have also resulted in increased interstitial fibrosis as described [14]. However, the timing of our analyses after 4 weeks allowed us to reproduce a situation, similar to the one found in humans with ST-elevation myocardial infarction, in which elevated sTWEAK levels indicated a worsened outcome 4 weeks after myocardial infarction [20].

Interestingly, Pachel et al. recently reported increased mortality in mice treated with albumin-conjugated TWEAK (HSA-Flag-TWEAK) [30]. The authors found an increased incidence of ventricular rupture in mice treated with HSA-Flag-TWEAK compared to control mice. Conversely, left ventricular function was not significantly changed during the first 3 days after MI. However, when neutrophils were depleted in HSA-Flag-TWEAK-treated mice, mortality remained unaffected despite reduced incidence of cardiac rupture. Thus, the findings by Pachel et al. fit to our findings in several ways: first, increased mortality was observed when mice were treated with TWEAK/HSA-Flag-TWEAK in both studies, even if myocardial rupture was prevented by neutrophil depletion. Second, left ventricular function was reduced in our study late after 28 days, whereas Pachel et al. only reported early functional evaluation up to 3 days after MI. And third, the lack of ventricular rupture in our study can be explained by the differences in the serum half-life of TWEAK and HSA-Flag-TWEAK. With albumin-conjugation, TWEAK remains significantly longer in the circulation thus aggravating the effects of TWEAK.

Although our results observed in mice after myocardial infarction are similar to the findings in the setting of human myocardial infarction, they are—like all cytokine studies—limited by species differences. In fact, many cytokine studies in the past have been shown to be of limited use in the human setting, due to species-specific mediators, targets, and signalling cascades. Thus—although our study using human recombinant sTWEAK provides a potential explanation of why elevated sTWEAK levels in humans with STEMI might be associated with adverse short-term outcome—they cannot be extrapolated to human pathophysiology. In addition, the nonreperfused myocardial infarction model of the mouse does not entirely fit into human pathophysiology.

Finally, TWEAK levels have been shown to be downregulated in the chronic setting, for example, in patients with chronic stable heart failure [19, 31]. The obvious discrepancy between acute and chronic stages of heart failure cannot be easily explained but might be related to an increased clearance of sTWEAK from the circulation, for example, by its known scavenger receptor CD163 on monocytes [32]. On the other hand, it is also possible that the reduced sTWEAK levels observed in the chronic setting are the result of successive downregulation with prolonged tissue injury. Nevertheless, additional research is necessary to address these obvious differences between chronic and acute stages of heart failure.

## 5. Conclusions

Our data show that sTWEAK in the setting of experimental myocardial infarction promotes left ventricular dysfunction. However, further research is necessary to exploit the role of the TWEAK-Fn14 axis in other models of cardiovascular pathology, such as ischemia reperfusion or aortic banding. In addition, further experimental settings are necessary to elucidate whether the protective effect of inhibition of intrinsic TWEAK or its receptor Fn14 has a potential to be exploited therapeutically in the future.

## Supplementary Material

Table 1: shoes the primer sequences used for quantitative RT-PCR.
Table 2: shows all antibodies used in western blot analysis.
Figure 1: shows echocardiographic parameters, heart weight/body weight ratio and expression of hypertrophy markers BNP and *β*-MHC.

## References

[1] Han S, Yoon K, Lee K (2003). TNF-related weak inducer of apoptosis receptor, a TNF receptor superfamily member, activates NF-kappa B through TNF receptor-associated factors. *Biochemical and Biophysical Research Communications*.

[2] Kleinbongard P, Heusch G, Schulz R (2010). TNF-alpha in atherosclerosis, myocardial ischemia/reperfusion and heart failure. *Pharmacology and Therapeutics*.

[3] Aukrust P, Ueland T, Müller F (1998). Elevated circulating levels of C-C chemokines in patients with congestive heart failure. *Circulation*.

[4] Behr TM, Wang X, Aiyar N (2000). Monocyte chemoattractant protein-1 is upregulated in rats with volume-overload congestive heart failure. *Circulation*.

[5] Chung ES, Packer M, Lo KH, Fasanmade AA, Willerson JT (2003). Randomized, double-blind, placebo-controlled, pilot trial of infliximab, a chimeric monoclonal antibody to tumor necrosis factor-*α*, in patients with moderate-to-severe heart failure: results of the anti-TNF therapy against congestive heart failure (ATTACH) trial. *Circulation*.

[6] Cuenca J, Goren N, Prieto P, Martín-Sanz P, Boscá L (2007). Selective impairment of nuclear factor-kappa B-dependent gene transcription in adult cardiomyocytes: relevance for the regulation of the inflammatory response in the heart. *The American Journal of Pathology*.

[7] Damås JK, Eiken HG, Øie E (2000). Myocardial expression of CC- and CXC-chemokines and their receptors in human end-stage heart failure. *Cardiovascular Research*.

[8] Deswal A, Bozkurt B, Seta Y (1999). Safety and efficacy of a soluble P75 tumor necrosis factor receptor (Enbrel, etanercept) in patients with advanced heart failure. *Circulation*.

[9] Levine B, Kalman J, Mayer L, Fillit HM, Packer M (1990). Elevated circulating levels of tumor necrosis factor in severe chronic heart failure. *The New England Journal of Medicine*.

[10] Hamid T, Gu Y, Ortines RV (2009). Divergent tumor necrosis factor receptor-related remodeling responses in heart failure: role of nuclear factor-kappa B and inflammatory activation. *Circulation*.

[11] Dogra C, Changotra H, Mohan S, Kumar A (2006). Tumor necrosis factor-like weak inducer of apoptosis inhibits skeletal myogenesis through sustained activation of nuclear factor-kappa B and degradation of MyoD protein. *The Journal of Biological Chemistry*.

[12] Dogra C, Changotra H, Wedhas N, Qin X, Wergedal JE, Kumar A (2007). TNF-related weak inducer of apoptosis (TWEAK) is a potent skeletal muscle-wasting cytokine. *The FASEB Journal*.

[13] Dogra C, Hall SL, Wedhas N, Linkhart TA, Kumar A (2007). Fibroblast growth factor inducible 14 (Fn14) is required for the expression of myogenic regulatory factors and differentiation of myoblasts into myotubes: evidence for TWEAK-independent functions of Fn14 during myogenesis. *The Journal of Biological Chemistry*.

[14] Feng SL, Guo Y, Factor VM (2000). The Fn14 immediate-early response gene is induced during liver regeneration and highly expressed in both human and murine hepatocellular carcinomas. *The American Journal of Pathology*.

[15] Burkly LC, Michaelson JS, Zheng TS (2011). TWEAK/Fn14 pathway: an immunological switch for shaping tissue responses. *Immunological Reviews*.

[16] Winkles JA (2008). The TWEAK-Fn14 cytokine-receptor axis: discovery, biology and therapeutic targeting. *Nature Reviews Drug Discovery*.

[17] Chorianopoulos E, Heger T, Lutz M (2010). FGF-inducible 14-kDa protein (Fn14) is regulated via the RhoA/ROCK kinase pathway in cardiomyocytes and mediates nuclear factor-kappa B activation by TWEAK. *Basic Research in Cardiology*.

[18] Jain M, Jakubowski A, Cui L (2009). A novel role for tumor necrosis factor-like weak inducer of apoptosis (TWEAK) in the development of cardiac dysfunction and failure. *Circulation*.

[19] Chorianopoulos E, Rosenberg M, Zugck C, Wolf J, Katus HA, Frey N (2009). Decreased soluble TWEAK levels predict an adverse prognosis in patients with chronic stable heart failure. *European Journal of Heart Failure*.

[20] Chorianopoulos E, Jarr K, Steen H, Giannitsis E, Frey N, Katus HA (2010). Soluble TWEAK is markedly upregulated in patients with ST-elevation myocardial infarction and related to an adverse short-term outcome. *Atherosclerosis*.

[21] Shi J, Jiang B, Qiu Y (2013). PGC1*α* plays a critical role in TWEAK-induced cardiac dysfunction. *PLoS ONE*.

[22] Sato S, Ogura Y, Mishra V (2013). TWEAK promotes exercise intolerance by decreasing skeletal muscle oxidative phosphorylation capacity. *Skeletal Muscle*.

[23] Campbell S, Burkly LC, Gao HX (2006). Proinflammatory effects of Tweak/Fn14 interactions in glomerular mesangial cells. *Journal of Immunology*.

[24] Jakubowski A, Ambrose C, Parr M (2005). TWEAK induces liver progenitor cell proliferation. *The Journal of Clinical Investigation*.

[25] Planavila A, Laguna JC, Vázquez-Carrera M (2005). Nuclear factor-*κ*B activation leads to down-regulation of fatty acid oxidation during cardiac hypertrophy. *The Journal of Biological Chemistry*.

[26] Mustonen E, Säkkinen H, Tokola H (2010). Tumour necrosis factor-like weak inducer of apoptosis (TWEAK) and its receptor Fn14 during cardiac remodelling in rats. *Acta Physiologica*.

[27] Schilling J, Lai L, Sambandam N, Dey CE, Leone TC, Kelly DP (2011). Toll-like receptor-mediated inflammatory signaling reprograms cardiac energy metabolism by repressing peroxisome proliferator-activated receptor *γ* coactivator-1 signaling. *Circulation*.

[28] Arany Z, Novikov M, Chin S, Ma Y, Rosenzweig A, Spiegelman BM (2006). Transverse aortic constriction leads to accelerated heart failure in mice lacking PPAR-*γ* coactivator 1*α*. *Proceedings of the National Academy of Sciences of the United States of America*.

[29] Finck BN, Kelly DP (2007). Peroxisome proliferator–activated receptor-*γ* coactivator-1 (PGC-1) regulatory cascade in cardiac physiology and disease. *Circulation*.

[30] Pachel C, Mathes D, Bayer B (2013). Exogenous administration of a recombinant variant of TWEAK impairs healing after myocardial infarction by aggravation of inflammation. *PLoS ONE*.

[31] Richter B, Rychli K, Hohensinner PJ (2010). Differences in the predictive value of tumor necrosis factor-like weak inducer of apoptosis (TWEAK) in advanced ischemic and non-ischemic heart failure. *Atherosclerosis*.

[32] Bover LC, Cardó-Vila M, Kuniyasu A (2007). A previously unrecognized protein-protein interaction between TWEAK and CD163: potential biological implications. *Journal of Immunology*.

