# Supplementary material for: TNF-Like Weak Inducer of Apoptosis Aggravates Left Ventricular Dysfunction after Myocardial Infarction in Mice
Source: Mediators Inflamm. 2014 Feb 20;2014:131950. doi: 10.1155/2014/131950 (PMC3945977; doi:10.1155/2014/131950)
Supplement: Supplementary file 1 — Table 1: shoes the primer sequences used for quantitative RT-PCR. Table 2: shows all antibodies used in western blot analysis. Figure 1: shows echocardiographic parameters, heart weight/body weight ratio and expression of hypertrophy markers BNP and β-MHC. [file 131950.f1.pdf]

Suppl. Table 1:

18S rRNA_for	5'-TCA AGA ACG AAA GTC GGA GG-3'
18S rRNA_rev	5'-GGA CAT CTA AGG GCA TCA C-3'
mouse_atp5o_for	5'-TCA TTT GGG TTT GAC CTA CAG CCG-3'
mouse_atp5o_rev	5'-AGA CCT GAA CAG GGG GCC TTA C-3'
mouse_β-MHC_for	5'-GCC AAC ACC AAC CTG TCC AAG TTC-3'
mouse_β-MHC_rev	5'-TGC AAA GGC TCC AGG TCT GAG GGC-3'
mouse_BNP_for	5'-ATC TCC TGC AGG TGC TGT CCC AG-3'
mouse_BNP_rev	5'-GGT CTT CCT ACA ACA ACT TCA GTG CGT TAC-3'
mouse_cox5b_for	5'-TGC TCA GCC TGT TCC CGG AAG T-3'
mouse_cox5b_rev	5'-CAT CAG TGG GGA CAC CAC CTC CA-3'
mouse_cycs_for	5'-CCA CGG TCT GTT CGG GCG G-3'
mouse_cycs_rev	5'-CCT CTC CCC AGG TGA TGC CTT TGT-3'
mouse_Fn14_for	5'-GTC TTG GGA TTC GGC TTG-3'
mouse_Fn14_rev	5'-GCA GAA GTC GCT GTG TGG T-3'
mouse_ndufb5_for	5'-CTG CAG CGG GCT TCG GTC TC-3'
mouse_ndufb5_rev	5'-TGC CGC ACA GGA GCG ACA GT -3'
mouse_PGC-1α_for	5'-CCC GTG GAT GAA GAC GGA TTG-3'
mouse_PGC-1α_rev	5'-GTG GGT GTG GTT TGC TGC ATG-3'
mouse_PPARα_for	5'-AGC TGG TGT AGC AAG TG-3'
mouse_PPARα_rev	5'-TCT GCT TTC AGT TTT GCT TT-3'
mouse_TWEAK_for	5'-AGG AGG AGC TGA CAG-3'
mouse_TWEAK_rev	5'-CCT CAT AAT GGG CTG-3'

rat_atp5o_for	5’-TGT CCC GAC AGG TGC GGA GT-3’
rat_atp5o_rev	5’-CGC AGC AGA GTA CAG GGC GG-3’
rat_cox5b_for	5’-GTA GGC GCT TTG GCG GCT CA-3’
rat_cox5b_rev	5’-GGG ACA CCA CCT CCA GAA GCC A-3’
rat_cycs_for	5’-CTT GGG CTA GAG AGC GGG ACG-3’
rat_cycs_rev	5’-TTA AAT TCG GTC CGG GCT GGT CAA-3’
rat_ndufb5_for	5’-GCT TCG GTC GCC GCT CTG AC-3’
rat_ndufb5_rev	5’-GCA CAG GAG CGA CAG TCT TCG G-3’
rat_PGC-1α_for	5’-CAC CAA ACC CAC AGA GAA CAG-3’
rat_PGC-1α_rev	5’-GGT GAC TCT GGG GTC AGA-3’

Suppl. Table 2:

Akt Antibody	Cell Signaling, Danvers, USA
GSK-3β Antibody	Cell Signaling, Danvers, USA
P44/42 MAPK (Erk 1/2) Antibody	Cell Signaling, Danvers, USA
Phospho-Akt (Ser473) Antibody	Cell Signaling, Danvers, USA
Phospho-p44/42 MAPK (Thr202/Tyr204) Antibody	Cell Signaling, Danvers, USA
Phospho-GSK-3β (Ser9) Antibody	Cell Signaling, Danvers, USA
TWEAK Antibody	Santa Cruz, Heidelberg, Germany
Fn14 Antibody	Santa Cruz, Heidelberg, Germany
PGC-1α Antibody	abcam, Cambridge, UK
Phospho-NFkB-p65 (Ser536) Antibody	Cell Signaling, Danvers, USA
α-Tubulin Antibody	Sigma-Aldrich, München, Germany

Suppl. Fig 1

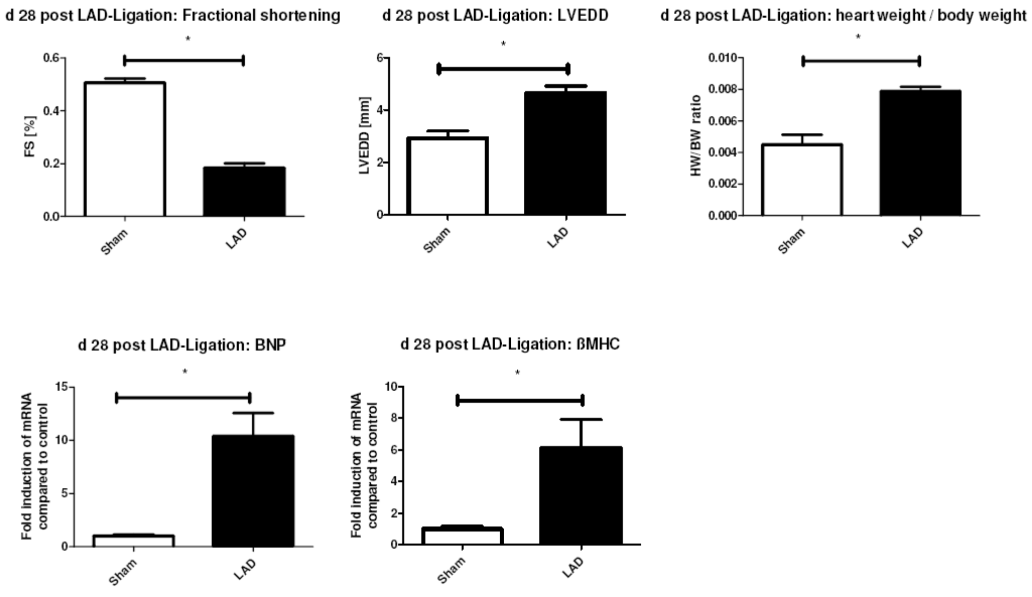
d 28 post LAD-Ligation: Fractional shortening
*
FS [%]
0.0
0.2
0.4
0.6
Sham
LAD
d 28 post LAD-Ligation: LVEDD
*
LVEDD [mm]
0
2
4
6
Sham
LAD
d 28 post LAD-Ligation: heart weight / body weight
*
HW/BW ratio
0.000
0.002
0.004
0.006
0.008
0.010
Sham
LAD
d 28 post LAD-Ligation: BNP
*
Fold induction of mRNA compared to control
0
5
10
15
Sham
LAD
d 28 post LAD-Ligation: ßMHC
*
Fold induction of mRNA compared to control
0
2
4
6
8
10
Sham
LAD
